# Investigating Pediatricians’ Practice, Knowledge, and Barriers in Diagnosing Cerebral Palsy

**DOI:** 10.3390/children12091274

**Published:** 2025-09-22

**Authors:** Vivian Wong, Stacey D. Miller, Olivia Scoten, Mor Cohen-Eilig, Stephanie Glegg, Angie Ip, Chetna Jetha, Kishore Mulpuri, Maureen O’Donnell, Ram Mishaal

**Affiliations:** 1 Rehabilitation Sciences Graduate Program, Faculty of Medicine, University of British Columbia, Vancouver, BC V6T 1Z3, Canada; 2 BC Children’s Hospital Research Institute, Vancouver, BC V5Z 4H4, Canada; smiller4@cw.bc.ca (S.D.M.);; 3 Department of Orthopaedic Surgery, BC Children’s Hospital, Vancouver, BC V6H 3V4, Canada; 4 Department of Physical Therapy, University of British Columbia, Vancouver, BC V6T 1Z3, Canada; 5 Department of Orthopaedics, University of British Columbia, Vancouver, BC V5Z 1M9, Canada; 6 Faculty of Medicine, University of British Columbia (UBC), Vancouver, BC V6T 1Z4, Canada; 7 Sunny Hill Health Centre for Children, Vancouver, BC V6H 3N1, Canada; 8 Department of Pediatrics, University of British Columbia, Vancouver, BC V6H 3V4, Canada; 9 Department of Occupational Science and Occupational Therapy, University of British Columbia, Vancouver, BC V6T 2B5, Canada; 10 Royal London Hospital, Barts Health NHS Trust, London E1 1FR, UK

**Keywords:** cerebral palsy, diagnosis, knowledge translation

## Abstract

**Highlights:**

**What are the main findings?**

- Not all pediatricians routinely encounter or diagnose children with cerebral palsy (CP) in their practice.
- Less than two-thirds of pediatricians reported diagnosing children with CP despite believing that early diagnosis is important.

**What is the implication of the main finding?**

- Pediatricians have identified gaps in knowledge, skills, and confidence in diagnosing CP in their practice.
- General pediatricians expressed a desire for a multidisciplinary or collaborative approach to support early diagnosis of CP.

**Abstract:**

**Background/Objectives:** Data from the Canadian Cerebral Palsy (CP) Registry suggests that children in British Columbia (BC) are diagnosed, on average, at 25 months of age. This is much later than currently recommended. This study aimed to examine current practices and beliefs of pediatricians in the province related to CP and CP diagnosis. **Methods:** All pediatricians and subspecialty pediatricians in the province were invited to participate in two consecutive online surveys. The initial survey aimed to assess current practice, knowledge of CP, and beliefs about diagnosis. The second survey, which was distributed to the same group of pediatricians, as well as pediatric neurologists and geneticists, aimed to re-assess current practice and identify specific barriers and facilitators to CP diagnosis. **Results:** The two surveys were completed by 76 and 59 respondents, respectively. Less than 60% of general pediatricians, in both surveys, reported diagnosing children with CP. In survey 2, only 50% of respondents felt that pediatricians should provide a diagnosis of CP. Most general pediatricians (93%) identified that pediatricians, with support from a developmental pediatrician or neurologist, should provide a diagnosis. Common barriers to an early CP diagnosis included uncertainty about other potential diagnoses and uncertainty over diagnosing at a young age. Lack of access to education and therapists to help inform the diagnosis were also frequently identified barriers. **Conclusions:** While general pediatricians are knowledgeable about CP, a significant proportion in those surveyed were not diagnosing CP, despite believing that early diagnosis is important. Findings from these surveys have identified that general pediatricians have gaps in knowledge, skills, and confidence in diagnosing CP. Support from a developmental pediatrician or neurology colleague was identified as a potential strategy to support earlier diagnosis.

## 1. Introduction

Cerebral palsy (CP) is the most common cause of physical disability in childhood, occurring at a prevalence of 1.6 per 1000 live births in high-income countries and higher in low- and middle-income countries [1]. CP is an umbrella term used to describe a group of permanent disorders in the development of movement and posture, causing activity limitation, that are attributed to a non-progressive disturbance in the developing fetal or infant brain [2].

Professional pediatric associations, government representatives, and community health advocates have long called for a change in practice to prioritize early assessment and diagnosis of CP [3,4,5]. An early diagnosis facilitates access to essential CP-specific interventions while neuroplasticity is greatest to maximize developmental outcomes [6]. Early diagnosis can also allow caregivers to regain control and develop active coping strategies, which can benefit parental mental health [7]. Feeling as though they were the last to know about a diagnosis can be particularly troubling for parents [8].

Despite available clinical guidelines and supportive evidence that a CP diagnosis can be made before 6 months’ corrected age, the mean age of diagnosis is 19 months in Canada [9] and, in the province of British Columbia (BC), this timeline lags even further at 25 months [10]. Children enrolled in the province’s hip surveillance programme with a diagnosis of ‘possible CP, not yet diagnosed’ had a mean age of 3.6 years [11]. Additionally, one in four children enrolled in the programme did not have a diagnosis of CP or possible CP, despite most meeting the criteria for CP [11]. There is no data pertaining to clinical practice of diagnosing CP in Canada or BC that could elucidate reasons for missing or delays in diagnosis. To achieve widescale implementation of best practice guidelines in early diagnosis of CP in BC, a baseline understanding of pediatricians’ current practices and knowledge of CP was needed.

In this two-phase project, the initial survey (survey 1) aimed to determine whether general pediatricians and subspecialty pediatricians in BC were actively diagnosing children with CP and to broadly assess knowledge and beliefs about CP and CP diagnosis. A second survey (survey 2) aimed to reassess current practices after the COVID-19 pandemic and identify specific barriers and facilitators to diagnosis in practice. Additionally, we aimed to determine learning needs for this population.

## 2. Materials and Methods

Invitations to participate in an anonymous open online survey were emailed to general and subspecialty pediatricians via provincial pediatric society mailing lists, academic departmental distribution lists, and professional associations in April 2018 (survey 1) and again in November 2022 (survey 2). The target population was practicing pediatricians across the province of British Columbia, Canada, including both general and subspecialist pediatricians. Trainees, including residents and pediatric fellows, and those retired from practice were excluded. Technical functionality was tested prior to distribution. The estimated length to complete each survey was 10–15 min. Ethical approval was obtained from the University of British Columbia Children’s and Women’s Research Ethics Board prior to survey dissemination. Respondents were invited to enter a draw covering registration costs of a local pediatric meeting, [$200 value (survey 1)] or two gift cards [$50 each (survey 2)].

For survey 1, participants were first asked six questions related to their years in practice, practice setting, caseload, and their current practices related to children with CP. To evaluate their knowledge of CP, those who reported treating children with CP were asked 11 true/false questions and asked to identify true causes of CP from a list of 12 possible etiologies of motor impairment. Respondents were asked if they were currently diagnosing CP in their practice and what type of clinicians they thought could make a definitive diagnosis of CP. Using a five-point Likert scale, pediatricians were then asked to rate their agreement with five statements related to diagnosis. Given that the importance of hip surveillance and that the lack of a CP diagnosis is perceived as a barrier to enrollment in the BC Provincial Hip Surveillance programme, four questions related to hip health were also included [12].

Survey 2 was distributed through the same channels to reach the same general and subspecialty pediatricians as survey 1. The invitation was also extended to child neurologists and medical geneticists as these subspecialities were identified as key providers of care to children with CP. Six demographic questions again asked about years in practice, practice setting, and caseload. Eight questions to assess CP knowledge were repeated from survey 1; two new true/false questions were added related to early diagnosis. Additional questions related to current practice of diagnosing CP (three questions), factors impacting provision of a diagnosis (three questions), confidence in providing a diagnosis with differing presentations (one question), and training in standardized assessment used in CP diagnosis (two questions) were included in survey 2. Questions and suggested answers in survey 2 were informed by the results of survey 1 and the Theoretical Domains Framework (TDF) [13]. Survey 2’s focus of inquiry was to comprehensively identify the behavioural determinants behind clinical practices related to assessment and diagnosis of CP. The TDF was selected to examine and identify the influences behind individual-level practice behaviours. Calls for the explicit use of theory in knowledge translation (KT) and implementation science studies have increased as the field continues to expand, particularly on what drives physician behaviour change [14,15,16,17]. The TDF consists of 14 domains, which are grouped determinants of behaviour from 33 behaviour change theories. TDF domains most relevant to clinical practice, such as knowledge, skills, beliefs about consequences, social and professional identity and role, and environmental context and resources informed question development.

Finally, respondents in both surveys were asked to identify the topics they were most interested in learning about, specific to the needs of children with CP (survey 1) and diagnosis and treatment of children with CP (survey 2), from a provided list. A five-point Likert scale was used in survey 1; respondents were asked to select their top three areas of interest in survey 2. The surveys are available as Supplementary Materials.

Given the exploratory nature of the surveys, only descriptive statistics were used for analysis of quantitative data. Categorical variables were described with frequencies and percentages. Continuous variables were described with means, where appropriate. Responses from partially completed surveys were included for analysis. For survey 2, two reviewers independently analyzed open-text responses using content analysis to further identify barriers and facilitators according to the TDF. Discrepancies were resolved with a third reviewer, the team’s implementation science researcher. To further understand differing perspectives on who could and should diagnose CP, subanalysis was performed on respondents who identified as practicing, in general, pediatrics only, or general pediatrics and another subspecialty. Those who specified developmental pediatrics and neurology were excluded in this group. 

## 3. Results

Surveys 1 and 2 were fully or partially completed by 76 and 59 respondents, respectively, using online tools. All responses were included in the analysis. An estimated 280 pediatricians received the survey 1 request for an estimated response rate of 27%. With the additional recruitment of pediatric neurologists and medical geneticists, the estimated response rate for survey 2 was 20%. A demographic summary of each sample survey is shown in Table 1.

A summary of responses to questions related to respondents’ care of children with CP are shown in Table 2. Most respondents in both surveys reported seeing six or fewer children with CP in an average month, suggesting children with CP are a small portion of most pediatricians’ caseloads.

Results of who respondents felt could (survey 1) and should (survey 2) provide a diagnosis are shown in Table 2. While 90% of respondents in survey 1 felt pediatricians can diagnose CP, only 50% of respondents in survey 2 felt that pediatricians should provide a diagnosis of CP. When this question was subanalyzed by respondents’ practice specialty, 43% of general pediatricians (*n* = 30), 60% of developmental pediatricians (*n* = 10), and 60% of neurologists (*n* = 5) felt pediatricians should provide a diagnosis. Most respondents, including 93% of general pediatricians, 80% of developmental pediatricians, and 100% of neurologists, felt that a pediatrician, with support from a developmental pediatrician or neurologist, should provide a diagnosis. Developmental pediatricians also identified that a diagnosis should be provided by a developmental pediatrician (90%) or a neurologist (80%). All of the neurologists felt a diagnosis should be provided by a developmental pediatrician (100%); four of five felt their discipline should also provide the diagnosis. General pediatricians were less likely to identify that these subspecialists (developmental pediatrician 67% and neurologist 70%) should provide a diagnosis. Finally, 77% of general pediatricians and 60% of both developmental pediatricians and neurologists felt that pediatricians with input from PT or OT should diagnose CP.

Responses to questions exploring CP knowledge level were similar across both surveys and are shown in Table 3. In considering potential causes of CP, genetic, chromosomal, metabolic, and maternal thyroid deficiency were least likely to be correctly identified across the two surveys. Knowledge of standardized tools for assessment was poor.

Table 4 shows levels of agreement with attitude statements related to diagnosis from survey 1 and confidence in decision making in different scenarios. Barriers to providing a diagnosis identified in survey 2 are shown in Table 5. The results assessing knowledge of potential causes of CP were consistent; respondents were most confident providing a diagnosis when children were born prematurely, had delayed development, spasticity, and/or periventricular leukomalacia on imaging (79%); without imaging results, confidence dropped (69%). Additionally, the most reported barriers were related to knowledge, confidence in decision making, and access to education or support.

Topics of greatest interest for education (combined Likert 4 and 5) in survey 1 included managing behavioural challenges, assessing and managing pain, diagnosing and managing sleep problems, and identifying children that require hip surveillance. Table 6 lists the identified learning needs by frequency of response in survey 2. The most requested topics included criteria for diagnosing a child with CP using a care pathway, and identification/management of tone in children with CP.

## 4. Discussion

Our surveys found that greater than one-third of pediatricians in the province were not diagnosing CP despite believing early diagnosis is important. While almost all respondents in the first survey felt pediatricians could provide a diagnosis (90%), when given the opportunity in survey 2 to specify who should be diagnosing children with CP, only 50% of respondents felt it should be pediatricians. Most of these respondents (87%) identified a ‘pediatrician, with support from developmental pediatrician or neurologist as needed’ as who should provide the diagnosis. Respondents also indicated this diagnosis should be provided by neurologists (72%), developmental pediatricians (71%), or pediatricians with support of physical or occupational therapists (69%). These results were consistent in the subanalysis between discipline groups of general pediatricians, developmental pediatricians, and neurologists. General pediatricians were less likely to identify that a diagnosis should be provided by a developmental pediatrician or a neurologist, suggesting they feel that general pediatricians should diagnose, but that support is required. Interestingly, the perception of parents’ dismissal of a general pediatrician’s opinion or their preference for the diagnosis to be given by a subspecialist such as a neurologist or developmental pediatrician was a barrier described by some respondents. These findings support previous results that found pediatricians experience uncertainty in detecting and diagnosing motor delays in children [18] and suggests a desire for practice support in clinical decision making.

Well-documented physician barriers to early diagnosis of CP include a lack of understanding of CP’s definition, fear of misdiagnosis, and preference to rule out other diagnoses while they “wait and see” if motor warning signs resolve on their own [19]. Aligned with the literature, pediatricians reported uncertainty about the possibility of motor delay originating from other diagnoses as a barrier to providing a diagnosis (Table 5) and we found weak knowledge of potential underlying etiologies of CP. While most pediatricians correctly identified that CP could result from periventricular leukomalacia, infections, brain malformations, or be of unknown etiology, fewer identified that chromosomal or other genetic conditions may be the underlying cause of CP (Table 3). These findings echo a recent survey of 330 physicians in the United States and Canada which found approximately 40% of respondents reported a genetic etiology as a reason to not diagnose CP in a child with a non-progressive motor disability [20]. However, recent findings now suggest that a potential genetic etiology can be identified in 10–30% of children with CP [21] and a consensus statement published by the International Genomic Consortium stated “identifying genetic etiologies or any other specific etiology should not change the clinical diagnosis of cerebral palsy” [22]. When asked in survey 2 about their confidence in making a diagnosis in different clinical situations, pediatricians were the least confident when a child presented with low tone and was born either term or preterm. This finding is, again, consistent with findings from Aravamuthan et al. [20].

Hesitancy existed amongst respondents to diagnose at a young age (Table 4), corresponding with almost 40% of respondents reporting they were diagnosing older children between the ages of three and five (Table 2). Qualitative comments in survey 2 highlight that the lack of perceived value of a CP diagnosis, particularly when a child was already receiving early intervention services, may be a barrier to early diagnosis. This belief by physicians that a CP diagnosis is not necessary contradicts parents’ desire for an early diagnosis documented in past research findings [23]. reported that all parents in their study, which investigated the experiences of providers and parents of children with CP with regard to early detection and intervention, stated that early diagnosis of CP or being informed about a high likelihood for CP was beneficial, compared with only 50% of providers believing so. In a recent survey of parents in our province, parents wanted less waiting for a diagnosis when asked what could have been performed to make obtaining the diagnosis easier [24]. Delayed diagnosis has been associated with greater rates of parental depression, stress, and dissatisfaction [8].

A diagnosis of CP, or the clinical designation of a high risk of CP, can be made accurately for a child as early as six months old [4]. A combination of validated motor and neurological assessments, along with magnetic resonance imaging (MRI), have been proven to be highly accurate for the prediction of CP [4]. The recommended standardized assessments include the Hammersmith Infant Neurological Examination (HINE), Prechtl Qualitative Assessment of General Movements (GMA), Test of Infant Motor Performance (TIMP), Alberta Infant Motor Scales (AIMS), Development Assessment of Youth and Children (DAYC), Neuro Sensory Motor Development Assessment (NSMDA), and Motor Assessment of Infants (MAI) [4]. In survey 2, we assessed familiarity with three of these tools and found that over 60% of respondents were not familiar with any (Table 3). Only 35% were familiar with or trained to use the HINE, 22% for the GMA, and 14% for the TIMP. Those who were familiar with these tools were exclusively subspecialists, such as developmental pediatricians, neonatologists, neurologists, and complex care specialists. The lack of familiarity with these standardized assessments for CP diagnosis amongst pediatricians points to a critical gap in education and training. However, given how few children with CP general pediatricians reported seeing in their practice, this finding is not surprising. The vast geography of BC and the proportion of the population living in rural and remote areas make it unlikely that this expertise is needed for pediatricians in all communities. Multidisciplinary teams, including those with pediatric occupational therapists or physiotherapists, may be best positioned to conduct and interpret these standardized assessments and to provide the diagnosis to families [4].

Most general pediatrician respondents in this survey reported seeing a small number of children with CP. Over 80% of respondents in our initial survey reported that 10% or less of their practice represented children with CP. In both surveys, almost 40% of respondents reported seeing one or fewer children with CP in an average month. These findings demonstrate that pediatricians provide care to children and youth with a range of conditions, consistent with other study findings [25]. However, well-baby checks of those born term or premature, and assessment of developmental delay, are common reasons for visits to the pediatrician [26,27]. As such, pediatricians should expect to encounter children with CP risk factors and their parents seeking a diagnosis. In 2020, an international expert panel published a list of clinical features that should prompt referral, as well as ‘warning signs’ that require monitoring [27]. Such tools help de-implement the “wait and see” approach. Of note, while in some jurisdictions pediatricians are primary care providers, in others, including in our province, they are not. A referral is required and, as with any specialist referral, this process often means significant wait times. This process results in an additional hurdle to timely diagnosis for children who present clinical risk factors later in infancy or childhood.

These studies have identified opportunities for improvements in medical education and healthcare services delivery, particularly towards team-based care as indicated by respondents’ desire for multidisciplinary support in detection and diagnosis. Improvements will also align with parental expectations for prompt assessment and communication about their child’s diagnosis. In addition to CP causes, risk factors, and detection, pediatric education may benefit from incorporation of life course theory and Developmental Origins of Health and Disease (DOHaD) that explain prenatal–postnatal disease pathways in humans, neural exposome concepts, structural, social, environmental determinants of health, and intersectionality to improve preventive care and early intervention in neurodevelopmental disorders [28,29]. With the growing emphasis on wellness and preventive medicine prior to presentation of disease phenotypes, including motor delays present in CP, education for maternal and pediatric providers across disciplines should consider structural, social, and environmental drivers of health.

This study has limitations. These surveys of pediatricians and subspecialists in one province may not be representative of healthcare settings in other parts of Canada. Bias may exist in the responses received; pediatricians with a greater interest in children with CP may have been more likely to respond. Importantly, our results may not be representative of pediatrician experiences in low- and middle- income countries that have a higher burden of disease and fewer resources [30]. While we aimed to include medical geneticists in survey 2, only two completed the survey, which limited our ability to conduct subgroup analysis. With greater knowledge of genetic causes of CP and increased access to genetic testing, further research on the role geneticists and genetic counsellors play in CP diagnosis is required. It is now known that genetic and epigenetic pathways, with interactions between genetic and environmental factors, affect fetal brain development, especially in the early gestational period [31,32,33,34,35]. These pathways may be further impacted by reproductive and pregnancy health factors, such as maternal disorders, socioeconomic barriers, mental health, and lifestyle factors. Future studies should inquire about healthcare providers’ knowledge and perspectives on these interactions. The design of survey 2 may have introduced biases to respondents. Survey questions and suggested answers were based on the research team’s selection of known or suspected TDF domains that influence pediatricians’ practice behaviours. This approach could have introduced over-emphasis on some TDF domains over others, such as knowledge, because of our perceived importance of those suspected barriers. Furthermore, whether or not the reported barriers and facilitators are the actual determinants (i.e., they have been experienced or encountered) or perceived (i.e., they are hypothetical barriers and enablers) remains. The perceived importance of particular factors may not always correspond with actual importance [18]. Establishing the reliability and validity of the surveys prior to their use may have reduced this uncertainty.

## 5. Conclusions

These surveys have illustrated that pediatricians have identified gaps in knowledge, skills, and confidence to diagnose CP in their practice. Responses suggest a lack of consensus on the roles of general pediatricians and pediatric subspecialists in diagnosing CP. All pediatricians can support early diagnosis of CP by recognizing early warning signs, performing prompt assessments, and referring to a subspecialist when needed. The divergence between pediatricians’ perspectives on capability and responsibility of diagnosis should not impede the process of detection and provision of early intervention for children at risk. Our findings illustrate the need for policy strategies such as a health services framework or mandate for providers, specific to local systems’ context, to support wider implementation of early CP diagnostic best practices. To achieve early diagnosis for all children at risk of CP in BC, the next steps will involve development and implementation of a KT intervention study to increase general pediatricians’ use of CP diagnosis guidelines in community settings.

## Figures and Tables

**Table 1:** Summary of pediatrician characteristics and caseload from survey 1 and 2.

Demographic Data (Survey 1, *n* = 76; Survey 2, *n* = 59)	Survey 1 (No., %)	Survey 2 (No., %)
Specialty *		
General Pediatrics	42 (55.3)	35 (59.3)
Neonatology	6 (7.9)	12 (20.3)
Developmental Pediatrics	4 (5.3)	10 (17.0)
Emergency Medicine	5 (6.5)	1 (1.7)
Hematology, Oncology, and BMT	5 (6.6)	0
Rheumatology	4 (5.3)	0
Critical Care	3 (3.9)	6 (10.2)
Complex Care	3 (3.9)	14 (23.7)
Neurology	0	6 (10.2)
Genetics	0	2 (3.4)
Other	9 (11.8)	5 (8.5)
Year pediatric or pediatric subspecialty training completed		
Prior to 1980	3 (3.9)	1 (1.7)
1980–1989	7 (9.2)	2 (3.4)
1990–1999	23 (30.3)	13 (22.4)
2000–2009	18 (23.7)	15 (25.9)
2010 to present	24 (31.6)	27 (46.6)
Unknown	1 (1.3)	1 (1.7)
Practice Setting		
Metro (>190,000)	47 (61.8)	36 (61.0)
Urban/Rural (40,001–190,000)	22 (28.9)	21 (35.6)
Rural (10,001–40,000)	6 (7.9)	1 (1.7)
Remote (<10,000)	1 (1.3)	1 (1.7)

Number of survey respondents (*n*); number of respondents selecting response (No.); bone marrow transplant (BMT). * Respondents had the option to select more than one specialty for area of work.

**Table 2:** Summary of practice questions related to children with CP from surveys 1 and 2.

	Survey 1 (No., %)	Survey 2 (No., %)
Estimated percentage of children in your practice who have CP (survey 1, *n* = 62)		
0%	14 (18.4)	n/a
1–10%	48 (63.2)	n/a
11–20%	7 (9.2)	n/a
21–50%	5 (6.6)	n/a
51–100%	2 (2.6)	n/a
Estimated number of children with CP seen in avg month (survey 1, *n* = 62; survey 2, *n* = 60)		
0	6 (9.7)	1 (1.7)
0–1	20 (32.3)	21 (35.0)
2–6	25 (40.3)	26 (43.3)
6–10	7 (11.3)	3 (5.0)
10+	4 (6.5)	9 (15.0)
Are you currently diagnosing CP?		
Yes (all respondents) (survey 1, *n* = 57; survey 2, *n* = 59)	32 (56.1)	38 (64.4)
Yes, (general pediatricians (survey 1, *n* = 46; survey 2, *n* = 31)	27 (58.7)	18 (58.0)
At what age do you typically diagnose CP (survey 2, *n* = 32)		
0–2 years	n/a	23 (71.9)
3–5 years	n/a	12 (37.5)
6–9 years	n/a	0
10+ years	n/a	0
What type of clinician can (survey 1)/should (survey 2) be responsible for making a diagnosis of CP? (select all) (survey 1 *n* = 57, survey 2, *n* = 55)		
Pediatrician	51 (89.5)	28 (50.9)
Pediatrician, with support from developmental pediatrician or neurologist as needed	n/a	48 (87.3)
Pediatrician, with input from PT or OT	n/a	38 (69.1)
Neurologist	56 (98.2)	40 (72.3)
Developmental Pediatrician	53 (93.0)	39 (70.9)
Orthopedic surgeon	26 (45.6)	11 (20.0)
General Practitioner	18 (31.6)	2 (3.6)
General practitioner, with support from pediatrician, developmental pediatrician, neurologist as needed	n/a	9 (16.4)
Other	4 (7.0)	1 (1.8)

Average (avg); not applicable (n/a); physical therapist (PT); occupational therapist (OT).

**Table 3:** Responses to knowledge questions involving cerebral palsy diagnosis from survey 1 and 2.

	Number Correctly Answered (*n*, %)	
	Survey 1 (*n* = 58)	Survey 2 (*n* = 50)
Knowledge Questions: True/False		
Abnormal brain imaging is required for a diagnosis of CP (F)	54 (93.1)	45 (90.0)
CP is an umbrella term that is not defined by etiology (T)	52 (91.4)	43 (86.0)
Of all children with CP, 40% are born prematurely and 60% are born at term (T)	39 (67.2)	17 (34.0)
Predicting severity of CP is most accurate after age 2 years (T)	50 (86.2)	27 (54.0)
A diagnosis of CP can only be made when the cause of the child’s motor impairment is known (F)	57 (98.3)	44 (88.0)
Evidence supports the early diagnosis of CP (T)	49 (84.5)	45 (90.0)
Clinical exam findings are poor indicator of hip displacement (T)	42 (72.4)	n/a
Risk of hip displacement increases from GMFCS level I to V (T)	55 (94.8)	n/a
Detection of hip displacement is completed through clinical and radiographical exams (T)	53 (96.6)	n/a
Pain always accompanies hip displacement (F)	57 (98.3)	n/a
A diagnosis of CP cannot be made before the age of 12 months (F)	n/a	35 (70.0)
A diagnosis of CP should be delayed since it increases the mental and emotional stress for caregivers (F)	n/a	45 (90.0)
Knowledge Questions: Yes/No		
Which underlying causes of motor impairment can result in CP?		
Genetic causes (Y)	42 (72.4)	37 (74.0)
Periventricular leukomalacia (Y)	56 (96.6)	47 (94.0)
Spinal nerve injury/spinal cord injury (N)	19 (67.2)	41 (82.0)
Acquired brain injury during the first 2–3 years of life (Y)	48 (82.8)	40 (80.0)
Chromosomal abnormality (Y)	41 (70.7)	36 (72.0)
Metabolic condition (Y)	43 (74.1)	36 (72.0)
Muscular origin (N)	15 (74.1)	44 (88.0)
Congenital cerebral malformation (Y)	50 (86.2)	45 (90.0)
Infection (meningitis/encephalitis) (Y)	51 (87.9)	43 (86.0)
Maternal thyroid deficiency (Y)	17 (70.7)	16 (32.0)
Unknown etiology (Y)	51 (87.9)	41 (82.0)
Perinatal brain injury (Y)	n/a	49 (98.0)
Intrauterine infection (Y)	n/a	45 (90.0)
Knowledge Questions: Are you familiar with or trained to use		
Hammersmith Infant Neurological Examination (HINE)	n/a	17 (34.7)
General Movements Assessment (GMA)	n/a	11 (22.5)
Test of Infant Motor Performance (TIMP)	n/a	7 (14.3)
I am not familiar or trained to use any assessments	n/a	30 (61.22)

True (T); False (F); Gross Motor Function Classification System (GMFCS); Yes (Y); No (N); Not assessed (n/a).

**Table 4:** Agreement with statements related to diagnosis of CP from survey 1 and 2.

		Responses (*n*, %)				
		1 (Not at All)	2	3	4	5 (Very Much)
Survey 1: Rate your level of agreement with the following statement (*n* = 57)						
It is more important to know the cause of a child’s motor impairment than to diagnose CP.		6 (10.5)	23 (40.4)	19 (33.3)	8 (14.0)	1 (1.8)
I prefer not to label children with a diagnosis of CP.		24 (42.1)	25 (43.9)	5 (8.8)	2 (3.5)	1 (1.8)
I do not think it is my role to diagnose children with CP.		24 (42.1)	14 (24.6)	8 (14.0)	7 (12.3)	4 (7.0)
Early diagnosis of CP is important for families so that diagnosis-specific treatments can be provided.		0 (0)	3 (5.3)	1 (1.8)	20 (35.1)	33 (57.9)
Survey 2: Rate your confidence making a diagnosis in the following situations (*n* = 48)						
Born premature, delayed development, spasticity		4 (8.3)	2 (4.2)	9 (18.8)	16 (33.3)	17 (35.4)
Born premature, delayed development, spasticity, PVL on imaging		2 (4.2)	4 (8.3)	4 (8.3)	12 (25.0)	26 (54.2)
Born premature, delayed development, low tone		4 (8.3)	20 (41.7)	12 (25.0)	9 (18.8)	3 (6.3)
Born term, delayed development, increased tone		3 (6.3)	13 (27.1)	13 (27.1)	13 (27.1)	6 (12.5)
Born term, delayed development, low tone		17 (35.4)	12 (25.0)	10 (20.8)	7 (14.6)	2 (4.2)
Survey 1 (*n* = 57) and 2 (*n* = 54): Rate your level of agreement with the following statement						
Providing a diagnosis of CP is important.	Survey 1	0 (0)	0 (0)	2 (3.5)	10 (17.5)	45 (78.9)
	Survey 2	0 (0)	0 (0)	5 (9.3)	10 (18.5)	39 (72.2)

(PVL: periventricular leukomalacia).

**Table 5:** Summary of barriers to early diagnosis identified in survey 2.

Grouped TDF Domains and Behaviour Determinants Impacting Pediatricians in Providing an Early CP Diagnosis (*n* = 50)	Responses (*n*, %)
Knowledge, skills, or confidence	
I am uncertain if the motor delay is due to other diagnoses	24 (48.0)
I feel uncertain making a diagnosis for children at a young age (e.g., under 2 years)	24 (48.0)
I am concerned about making a false positive diagnosis	18 (36.0)
I have difficulty recognizing early motor type, topography, and severity of CP	16 (32.0)
I do not have enough knowledge to make a diagnosis of CP	15 (30.0)
I do not know what supports are available for the child and family	13 (26.0)
I am not sure of the next steps after making a diagnosis	13 (26.0)
I prefer to monitor whether the motor delay will improve over time	11 (22.0)
I do not feel comfortable diagnosing when I am unable to classify a GMFCS level	6 (12.0)
I do not feel comfortable communicating this diagnosis to a family	5 (10.0)
Other	7 (14.0)
Not applicable	9 (18.0)
Environment or systems	
Access to education/training on CP diagnosis	21 (42.0)
Access to therapists (OT or PT) in my community to help inform the diagnosis	21 (42.0)
Time required or lack of time to learn about diagnosing CP	14 (28.0)
Length of appointment time	14 (28.0)
Delayed referrals to specialists due to child not having a family doctor	13 (26.0)
No colleagues with expertise/experience to review cases/consult	10 (20.0)
Lack of imaging services to support the diagnosis	9 (18.0)
My peers and colleagues currently are not providing CP diagnoses	8 (16.0)
Patient waitlist length	6 (12.0)
No systems in place to triage cases	5 (10.0)
Other	4 (8.0)
Not applicable	7 (14.0)
Other	
I feel the family wants a diagnosis from a neurologist, developmental pediatrician or other specialist	21 (42.0)
I prefer to refer to a specific specialist for diagnosis; I do not think it is my role to provide the diagnosis	9 (18.0)
It is not important to have a CP diagnosis in order to treat functionally	7 (14.0)
I do not think families are ready for the diagnosis and the burden it will place on them	4 (8.0)
I do not think it is important to provide a diagnosis of CP since it will not change the child’s outcome	4 (8.0)
I prefer not to use labels like CP	4 (8.0)
Other	7 (14.0)
Not applicable	14 (28.0)

Theoretical Domains Framework (TDF).

**Table 6:** Top learning interests related to children with cerebral palsy identified in survey 2.

Topic Area (by Domain)	Responses *n* (%)
Diagnosis	
Criteria for diagnosing a child with CP using a care pathway	30 (63)
Completing the required assessments related to tone and CP	24 (50)
Testing to determine the etiological cause of CP	16 (33)
Determining a child’s classification levels	11 (23)
The different types of CP	9 (19)
How to communicate a diagnosis of CP	9 (19)
Assessing other areas of development in the context of motor impairment	6 (13)
Treatment	
Assessing and managing tone	24 (50)
Next steps after making a diagnosis of CP	17 (35)
Assessing and managing pain in children with CP	15 (31)
Prognostication of future function after diagnosing CP	8 (17)
Identifying children that require hip surveillance	5 (10)
Diagnosing and managing sleep problems in children with CP	5 (10)
Managing nutrition and feeding problems in children with CP	5 (10)
Managing seizures in children with CP	3 (6)
Advising families on complementary and alternative medicines	2 (4)
Resources	
Providing families with resources	21 (44)
Providing family education on preventative healthcare	7 (15)
Other	2 (4)

## Data Availability

The data that support the findings of this study are available from the corresponding author upon reasonable request. Data are not publicly available due to privacy reasons.

## Supplementary Materials

Copies of the surveys can be downloaded at: https://www.mdpi.com/article/10.3390/children12091274/s1.
