# Supplementary material for: Investigating Pediatricians’ Practice, Knowledge, and Barriers in Diagnosing Cerebral Palsy
Source: Children (Basel). 2025 Sep 22;12(9):1274. doi: 10.3390/children12091274 (PMC12468520; doi:10.3390/children12091274)
Supplement: Supplementary file 1 [file children-12-01274-s001.zip › children-3843955-supplementary.pdf]

Supplementary Material: Questionnaires for Survey 1 and 2

SURVEY 1

Part 1:
We recognize that some of you will have great experience with children and youth with cerebral palsy and others are subspecialists who may have only occasional contact with children with cerebral palsy. Our first series of questions will help us understand your experience with children and youth with cerebral palsy and your practice needs.

Please answer the following questions about your practice and experience.

1. In what year did you complete your pediatric or pediatric subspecialty training?

2. Which of the following best describes your area of work?
   - Adolescent Health and Medicine.
   - Allergy and Immunology.
   - Biochemical Diseases.
   - Cardiology.
   - Critical Care.
   - Dermatology.
   - Developmental Pediatrics.
   - Emergency Medicine.
   - Endocrinology and Diabetes.
   - Gastroenterology, Hepatology and Nutrition.
   - General Pediatrics.
   - Hematology, Oncology and BMT.
   - Infectious Diseases.
   - Neonatology.
   - Nephrology.
   - Neurology.
   - Respiratory Medicine.
   - Rheumatology.
   - Translational Therapeutics.
   - Complex Care.
   - Social Pediatrics.

3. Which best describes your practice setting?
   Metro (population over 190,001).
   Urban/rural (population between 40,001 and 190,000).
   Rural (population between 10,001 and 40,000).

Remote (population between 0 and 10,000).

4. Please estimate the percentage of the children in your practice who have cerebral palsy:
   0%.
   1-10%.
   11-20%.
   21-30%.
   31-40%.
   41-50%.
   51-60%.
   61-70%.
   71-80%.
   81-90%.
   91-100%.

If you answered 0%, jump to question 23, with the following intro: You have identified that you do not see children with cerebral palsy in your practice. We have one final question related to your interest in learning about children with cerebral palsy.

5. Please estimate the number of children with cerebral palsy that you see in your clinic in an average month. (Consider the last month) [open text]

6. Recognizing that there will be variation, which of the following best describes the most common frequency at which you see your patients with cerebral palsy?
   - Annually.
   - Every 6 – 12 months.
   - Every 4 – 6 months.
   - Every 2 – 4 months.
   - Monthly.
   - More than monthly.

Part 2:
We would now like to start identifying your learning needs by asking about your understanding of cerebral palsy.

Please answer the following True and False questions about cerebral palsy.
7. Abnormal brain imaging is required for a diagnosis of cerebral palsy.
8. Cerebral palsy is an umbrella term that is not defined by etiology.

9. Children with cerebral palsy deteriorate over time.
10. Of all children with cerebral palsy, 40% are born prematurely and 60% are born at term.
11. Predicting severity of cerebral palsy is most accurate after age 2 years.
12. A diagnosis of cerebral palsy can only be made when the cause of the child's motor impairment is known.
13. Evidence supports the early diagnosis of cerebral palsy.
14. Clinical exam findings are a poor indicator of hip displacement.
15. The risk for hip displacement increases from GMFCS level I to V.
16. Pain always accompanies hip displacement.
17. Detection of hip displacement is completed through clinical and radiographical exams.

The following questions are related specifically to the diagnosis of cerebral palsy.

18. Which of the following underlying causes of motor impairment can result in cerebral palsy? [select all that apply]
    - Genetic cause.
    - Periventricular leukomalacia.
    - Perinatal brain injury.
    - Spinal nerve injury.
    - Acquired brain injury during the first 2-3 years of life.
    - Chromosomal abnormality.
    - Metabolic condition.
    - Muscular origin.
    - Cerebral malformation.
    - Infection (meningitis/encephalitis).
    - Maternal thyroid deficiency.
    - Unknown etiology.

19. Are you currently diagnosing children with cerebral palsy in your practice? [Yes or No]

20. Of the following providers, who do you think can make a definitive diagnosis of cerebral palsy? [select all that apply]
    - Pediatrician.
    - Developmental pediatrician.
    - Neurologist.
    - Family physician.
    - Orthopedic surgeon.
    - Other: _______________________________.

21. Please rate your agreement with the following statements: [1 to 5 Likert scale from not at all to very much so]
    a. Providing a diagnosis of cerebral palsy is important.
    b. It is more important to know the cause of a child's motor impairment than to diagnose cerebral palsy.
    c. I prefer not to label children with a diagnosis of cerebral palsy.
    d. I do not think it is my role to diagnose children with cerebral palsy.
    e. Early diagnosis of cerebral palsy is important for families so that diagnosis-specific treatments can be provided.
    f. I identify Gross Motor Function Classification System (GMFCS) levels for all of my patients with cerebral palsy.
    g. I think hip displacement in children with cerebral palsy is a problem that requires standardized monitoring.
    h. I am familiar with the Child Health BC Hip Surveillance Program for Children with Cerebral Palsy.
    i. I know where to find clinical guidelines and learning materials for the Child Health BC Hip Surveillance Program for Children with Cerebral Palsy.

Part 3: (had to take this part out)
The following questions are related to your current practice with children with cerebral palsy. Your answers to these questions will help inform the educational opportunities we will create.

22. Please rate **your interest** in learning about the following topics **specific** to the needs **of children with cerebral palsy as a means to increase your ability to provide care:** [1 to 5 Likert scale from not at all to very much so]
    a) Completing a physical exam.
    b) Diagnosing a child with cerebral palsy.
    c) Testing to determine the etiological cause of cerebral palsy.
    d) Assessing and treating feeding/swallowing difficulties.
    e) Assessing and treating growth and nutrition.
    f) Assessing development in the context of motor impairment.
    g) Identifying children that require hip surveillance.
    h) Identifying children with scoliosis.
    i) Assessing and treating bone health/osteopenia.
    j) Prescribing bracing (e.g., ankle foot orthotics).
    k) Assessing and managing tone.

l) Managing constipation.
m) Assessing vision and interpreting assessment results.
n) Assessing hearing and interpreting assessment results.
o) Assessing communication abilities and recommending referrals and interventions.
p) Managing seizures.
q) Diagnosing and managing sleep problems.
r) Assessing and managing pain.
s) Managing behavioral challenges.
t) Assessing self-help skills/adaptive behaviors.
u) Making referrals to appropriate community services (PT, OT, SLP, dietician).
v) Supporting learning needs.
w) Advising re: complementary and alternative medicines.
x) Determining a child's GMFCS level.
y) Determining a child's MACS level.
z) Determining a child's CFCS level.
aa) Providing education on preventative healthcare (e.g. special immunizations, dental care).

23. Thank you for your time. If you wish to be entered in a draw to win a registration for the 2018 BC Pediatric Society Annual Conference, please provide your email address. Results will be kept separate from email addresses. Email addresses will be kept confidential and will not be saved following the draw. [open text]

Thank you for your time.

SURVEY 2

Part 1 - We recognize that some of you will have experience with children and youth with cerebral palsy and others are subspecialists who may have only occasional contact with children with cerebral palsy. Our first series of questions will help us understand your experience with children and youth with cerebral palsy and your practice needs.

Please answer the following questions about your practice and experience.

24. In what year did you complete your residency or subspecialty training? [Drop-down menu with years]

25. Which of the following best describes your area(s) of work? (Select all that apply)
    - ❑ Complex Care.
    - ❑ Critical Care.
    - ❑ Developmental Pediatrics.
    - ❑ Emergency Medicine.
    - ❑ General Pediatrics.
    - ❑ Genetics.
    - ❑ Neonatology.
    - ❑ Neurology.
    - ❑ Other: _____________.

26. Which best describes your practice setting?
    - Metro (population over 190,001).
    - Urban/rural (population between 40,001 and 190,000).
    - Rural (population between 10,001 and 40,000).
    - Remote (population between 0 and 10,000).

27. In which health region(s) of BC do you work? (Select all that apply). Note: this question refers to the geographic region, not the specific health authority that may employ you.
    - ❑ Fraser Health.
    - ❑ Interior Health.
    - ❑ Island Health.
    - ❑ Northern Health.
    - ❑ Vancouver Coastal Health.

28. What type of clinical setting do you work in? (Check all that apply)
    - ❑ Hospital: inpatient.
    - ❑ Hospital: outpatient.
    - ❑ Community.
    - ❑ Other: ______________________

29. Please estimate the number of children per month you see in your practice who have cerebral palsy.
    - ❑ I do not see children with CP.
    - ❑ 0-1 (in some months I see one and in other months I see none).
    - ❑ 2-3.

- ❑ 4-6.
- ❑ 7-9.
- ❑ 10+.

If answer is "I do not see children with CP", message will appear: "You have identified that you do not see children with cerebral palsy in your practice. We have some final questions related to your interest in learning about working with children with cerebral palsy." Participants will answer questions 7-8,19-22.

Part 2 - We would now like to start identifying your learning needs by asking about your understanding of cerebral palsy.

30. Please answer the following True and False questions about cerebral palsy:
    - Abnormal brain imaging is required for a diagnosis of cerebral palsy.
    - Cerebral palsy is an umbrella term that is not defined by etiology.
    - Children with cerebral palsy deteriorate over time.
    - Of all children with cerebral palsy, 40% are born prematurely and 60% are born at term.
    - Predicting severity of cerebral palsy is most accurate after the age of two.
    - A diagnosis of cerebral palsy can only be made when the cause of the child's motor impairment is known.
    - Evidence supports the early diagnosis of cerebral palsy.
    - A diagnosis of CP cannot be made before the age of 12 months.
    - A diagnosis of CP should be delayed since it increases the mental and emotional stress for caregivers.

31. Which of the following underlying causes of motor impairment can result in cerebral palsy? (Select all that apply)
    - ❑ Periventricular leukomalacia.
    - ❑ Perinatal brain injury.
    - ❑ Spinal cord injury.
    - ❑ Acquired brain injury during the first two years of life.
    - ❑ Chromosomal abnormality.
    - ❑ Genetic causes.
    - ❑ Metabolic condition.
    - ❑ Muscular origin.
    - ❑ Congenital cerebral malformation.
    - ❑ Postnatal infection (meningitis/encephalitis).
    - ❑ Intrauterine infection.
    - ❑ Maternal thyroid deficiency.
    - ❑ Unknown etiology.

Part 3 - The following questions are related to your current practice with children with cerebral palsy. Your answers to these questions will help inform the educational opportunities we will create.

7. Are you currently diagnosing children with cerebral palsy in your practice? Yes/No.

Questions 8-10 will be displayed if respondent answered yes

8. How often do you diagnose children with CP per year? [open text]

9. Generally, at what age do you typically provide a diagnosis of CP?
   - ❑ 0-2 years.
   - ❑ 3-5 years.
   - ❑ 6-9 years.
   - ❑ 10+ years.

10. Do you provide resources to the family after the diagnosis? (Select all that apply)
    - ❑ Yes, information about what cerebral palsy is.
    - ❑ Yes, information about what the future may involve for their child.
    - ❑ Yes, information or referrals to support services and funding.
    - ❑ No; I would like to, but I am unaware of what is available.
    - ❑ No; I do not find resources to be helpful.

11. Please rate your agreement with the following statement: *Providing a diagnosis of cerebral palsy is important.*
    - I strongly agree.
    - I agree.
    - I am ambivalent.
    - I disagree.
    - I strongly disagree.

12. What type of clinician(s) do you think should be responsible for making a diagnosis of CP? (Select all that apply)
    - ❑ General practitioner.
    - ❑ General practitioner, with support from pediatrician/developmental pediatrician/neurologist as needed.
    - ❑ Pediatrician.
    - ❑ Pediatrician, with support from developmental pediatrician/neurologist as needed.
    - ❑ Pediatrician, with input from physiotherapists or occupational therapists.
    - ❑ Developmental pediatrician.
    - ❑ Neurologist.
    - ❑ Orthopedic surgeon.
    - ❑ Other: ___________________________.

13. Are there **<u>knowledge, skills, or confidence</u>** factors that impact you providing an early diagnosis of CP? (Select all that apply)
    - ❑ I do not have enough knowledge about how to make a diagnosis of CP.
    - ❑ I feel uncertain about making a diagnosis for children at a young age (e.g., under two years).
    - ❑ I am uncertain if the motor delay is due to other diagnoses.

- ❑ I prefer to monitor to determine if motor delay will improve over time.
- ❑ I have difficulty recognizing early motor type, topography, and severity of CP.
- ❑ I do not feel comfortable diagnosing when I am unable to classify a GMFCS (Gross Motor Function Classification System) level.
- ❑ I am concerned about making a false positive diagnosis.
- ❑ I do not feel comfortable communicating this diagnosis to a family.
- ❑ I am not sure of the next steps after making a diagnosis.
- ❑ I do not know what supports are available for the child and family.
- ❑ Other (specify): ___________________________.
- ❑ Not applicable.

14. Children with CP have different risk factors and clinical presentations that may influence diagnosis. Please rate your confidence in making a diagnosis in the following situations (anchors on 1=not at all and 5=very confident).
    - Born premature, delayed development, spasticity.
    - Born premature, delayed development, spasticity, periventricular leukomalacia (PVL) on imaging.
    - Born premature, delayed development, low tone.
    - Born term, delayed development, increased tone.
    - Born term, delayed development, low tone.

15. Are there **environmental or systems** factors, such as your practice setting, resources, funding, or policies, that impact you providing an early diagnosis of CP? (Select all that apply)
    - ❑ Length of appointment time (e.g., not enough time required to do an assessment).
    - ❑ Patient waitlist length (e.g., long waitlist prevents assessing a child in a timely manner).
    - ❑ Access to therapists (OT or PT) in my community to help inform the CP diagnostic process.
    - ❑ No systems in place in my practice to triage cases.
    - ❑ Lack of imaging services to support the diagnosis.
    - ❑ Delayed referrals to specialists due to child not having a family doctor.
    - ❑ No professional support or colleagues with expertise/experience to review cases/consult.
    - ❑ My peers and colleagues are not currently providing CP diagnoses.
    - ❑ Time required or lack of time to learn about diagnosing CP.
    - ❑ Access to education/training on CP diagnosis.
    - ❑ Other: _____________.
    - ❑ Not applicable.

16. Are there **other factors** that impact you providing a diagnosis of CP? (Select all that apply)
    - ❑ I prefer to refer to a specific specialist for diagnosis; I do not think it is my role to provide the diagnosis.
    - ❑ I feel the family wants a diagnosis from a neurologist, developmental pediatrician or other specialist.
    - ❑ It is not important to have a CP diagnosis in order to treat functionally.
    - ❑ I do not think families are ready for the diagnosis and the burden it will place on them.

- ❑ I do not think it is important to provide a diagnosis of CP since it will not change the child's outcome or if the child already has access to therapy or supports.
- ❑ I prefer not to use labels like CP.
- ❑ Other: ______________.
- ❑ Not applicable.

17. Are you familiar with or trained to use any of the following clinical assessments? (Select all that apply)
    - ❑ Hammersmith Infant Neurological Exam (HINE).
    - ❑ General Movements Assessment (GMA).
    - ❑ Test of Infant Motor Performance (TIMP).
    - ❑ I am familiar with or trained to use this other clinical assessment tool(s) to diagnose CP: ______________.
    - ❑ I am not familiar with or trained to use any clinical assessments to diagnose CP.

18. Which of the following clinical assessments are you actively using in your practice? (Select all that apply). *Question will only be displayed if respondent* ***does not choose*** *"I am not familiar with..."*
    - ❑ Hammersmith Infant Neurological Exam (HINE).
    - ❑ General Movements Assessment (GMA).
    - ❑ Test of Infant Motor Performance (TIMP).
    - ❑ I am using this other clinical assessment tool(s) to diagnose CP: ______________.

19. **To increase your knowledge and skills** specific to the needs of children with CP, please tell us the **top five topics about which you are most interested in** (Select up to five from the entire list):

    Diagnosis
    - ❑ Completing the required assessments related to tone and cerebral palsy (e.g., physical exam or HINE).
    - ❑ Criteria for diagnosing a child with CP using a care pathway.
    - ❑ The different types of CP.
    - ❑ Determining a child's classification levels, e.g., GMFCS, Manual Ability Classification System (MACS), Communication Function Classification System (CFCS).
    - ❑ Testing to determine the etiological cause of cerebral palsy.
    - ❑ Assessing other areas of development in the context of motor impairment.
    - ❑ How to communicate a diagnosis of CP.

    Treatment
    - ❑ Identifying children that require hip surveillance.
    - ❑ Assessing and managing tone.
    - ❑ Managing seizures in children with CP.
    - ❑ Diagnosing and managing sleep problems in children with CP.
    - ❑ Managing nutrition and feeding problems in children with CP.
    - ❑ Assessing and managing pain in children with CP.
    - ❑ Next steps after making a diagnosis of CP (e.g., early intervention, medical management, community referrals and other considerations).

- ❑ Prognostication of future function after diagnosing CP.
- ❑ Advising families on complementary and alternative medicines.

Resources

- ❑ Providing family education on preventative healthcare (e.g., special immunizations, dental care).
- ❑ Providing families with resources (e.g., information about CP, referrals to support services, financial resources).

❑ Other:________________.

20. What type of education/training best suits your learning needs? (Select all that apply)
    - o Virtual on-demand webinars.
    - o Virtual live webinars.
    - o In-person education/training opportunities.
    - o Self-paced online learning modules.
    - o Physician-to-physician consultation with a specialist, such as a developmental pediatrician.
    - o A community of practice (peers coming together to support learning needs).
    - o Mentorship within my discipline.
    - o Other ideas or suggestions: ___________________.

21. What would make it most feasible for you to take advantage of these education/training opportunities? [Open text]

22. Is there anything else you would like to tell us about diagnosing or caring for children with cerebral palsy in the community setting? [Open text]

Could we contact you about future activities of the project as it pertains to early diagnosis of cerebral palsy? If so, please leave your name and email. The contact information collected will only be used for this purpose.

Name:
Email:

To be entered into the prize draw for one of two $50 gas gift cards, please leave your name and email.

Name:
Email:
